# The pattern-recognition molecule H-ficolin in relation to diabetic kidney disease, mortality, and cardiovascular events in type 1 diabetes

**DOI:** 10.1038/s41598-021-88352-y

**Published:** 2021-04-26

**Authors:** Jakob Appel Østergaard, Fanny Jansson Sigfrids, Carol Forsblom, Emma H. Dahlström, Lena M. Thorn, Valma Harjutsalo, Allan Flyvbjerg, Steffen Thiel, Troels Krarup Hansen, Per-Henrik Groop

**Affiliations:** 1 Department of Endocrinology and Internal Medicine, Aarhus University Hospital, Aarhus, Denmark; 2 Steno Diabetes Center Aarhus, Aarhus University Hospital, Aarhus, Denmark; 3 Folkhälsan Institute of Genetics, Folkhälsan Research Center, Helsinki, Finland; 4 Abdominal Center, Nephrology, University of Helsinki and Helsinki University Hospital, Helsinki, Finland; 5 Research Program for Clinical and Molecular Metabolism, Faculty of Medicine, University of Helsinki, Helsinki, Finland; 6 Department of General Practice and Primary Health Care, University of Helsinki and Helsinki University Hospital, Helsinki, Finland; 7 National Institute for Health and Welfare, Helsinki, Finland; 8 Steno Diabetes Center Copenhagen, The Capital Region of Denmark, Copenhagen, Denmark; 9 Department of Biomedicine, Aarhus University, Aarhus, Denmark; 10 Department of Diabetes, Central Clinical School, Monash University, Melbourne, Australia

**Keywords:** Complement cascade, Predictive markers, Cardiovascular biology, Endocrine system and metabolic diseases, Predictive markers, Diabetes, Acute coronary syndromes, Chronic kidney disease, Kidney diseases, Renal replacement therapy, Comorbidities, Inflammation, Risk factors

## Abstract

H-ficolin recognizes patterns on microorganisms and stressed cells and can activate the lectin pathway of the complement system. We aimed to assess H-ficolin in relation to the progression of diabetic kidney disease (DKD), all-cause mortality, diabetes-related mortality, and cardiovascular events. Event rates per 10-unit H-ficolin-increase were compared in an observational follow-up of 2,410 individuals with type 1 diabetes from the FinnDiane Study. DKD progression occurred in 400 individuals. The unadjusted hazard ratio (HR) for progression was 1.29 (1.18–1.40) and 1.16 (1.05–1.29) after adjustment for diabetes duration, sex, HbA_1c_, systolic blood pressure, and smoking status. After adding triglycerides to the model, the HR decreased to 1.07 (0.97–1.18). In all, 486 individuals died, including 268 deaths of cardiovascular causes and 192 deaths of complications to diabetes. HRs for all-cause mortality and cardiovascular mortality were 1.13 (1.04–1.22) and 1.05 (0.93–1.17), respectively, in unadjusted analyses. These estimates lost statistical significance in adjusted models. However, the unadjusted HR for diabetes-related mortality was 1.19 (1.05–1.35) and 1.18 (1.02–1.37) with the most stringent adjustment level. Our results, therefore, indicate that H-ficolin predicts diabetes-related mortality, but neither all-cause mortality nor fatal/non-fatal cardiovascular events. Furthermore, H-ficolin is associated with DKD progression, however, not independently of the fully adjusted model.

## Introduction

Type 1 diabetes is a prevalent, chronic disease associated with several metabolic disturbances. Despite an optimized standard of care, the disease remains accompanied by excess morbidity and mortality, mostly mediated by its long-term micro- and macrovascular complications^1,2^.

The pattern-recognition molecules of the complement system, such as H-ficolin (also known as Ficolin-3) and mannan-binding lectin (MBL), bind specific molecular patterns that may be present on the surface of microorganisms and can thereby activate the lectin pathway of the complement system. As a consequence, an inflammatory reaction may evolve^3^. In the Steno Diabetes cohort in Denmark, we have previously shown that H-ficolin is associated with an increased risk of incident microalbuminuria in type 1 diabetes^4^, and MBL has been linked to diabetic kidney disease (DKD) in both type 1^5–7^ and type 2 diabetes^8^. A recent study compared all-cause mortality in individuals with type 1 diabetes grouped by genetically determined differences in MBL concentration and found a higher mortality rate among individuals with genotypes encoding high MBL concentrations – possibly suggesting a causal relationship^9^.

Hyperglycemia alters glycans enzymatically through the hexosamine pathway as well as non-enzymatically by the formation of advanced glycation end-products^10,11^. These glycan alterations are proposed to cause an adverse complement auto-attack initiated by pattern-recognition molecules. In line with this, MBL promotes diabetic kidney changes and accumulates in multiple tissues in experimental models of diabetes^12–16^.

Taken together, clinical and preclinical studies point towards an auto-attack from the complement system in diabetes, and the pattern-recognition molecules have emerged as potential culprits in the pathogenesis of late diabetic complications. In this study, we aimed to validate the previously observed association of H-ficolin concentration and incident microalbuminuria in a nationwide, multicenter Finnish cohort of individuals with type 1 diabetes. Additionally, we aimed to examine whether H-ficolin concentration is associated with the progression of DKD as well as with mortality and cardiovascular outcomes in a large and well-characterized population of individuals with type 1 diabetes.

## Results

### Baseline characteristics

This study included 2,410 individuals with type 1 diabetes from the Finnish Diabetic Nephropathy (FinnDiane) Study. The baseline clinical characteristics of the study participants, stratified by median H-ficolin concentration (31.18 μg/ml) at baseline, are summarized in Table 1. In brief, those in the group of higher H-ficolin concentration had also higher systolic and diastolic blood pressure, higher HbA_1c_, and were more often current or former smokers. There was also a clear difference in the sex distribution between the groups, as a larger proportion of those with higher H-ficolin were men. However, we did not observe any difference in mean age or diabetes duration. Individuals with high H-ficolin were more likely to have more advanced stages of DKD, whereas the difference in eGFR was not significant after correction for multiple testing.

**Table 1:** Baseline characteristics by median H-Ficolin concentration.

	H-ficolin < 31.18 μg/ml	H-ficolin ≥ 31.18 μg/ml	*P*	*P*_*19 tests*_
n	1205	1205		
Sex (woman)	711 (59.0%)	432 (35.9%)	< 0.001	< 0.001
Age (years)	36.7 (11.3)	37.5 (11.4)	0.11	1
Diabetes duration (years)	21.4 (12.1)	21.7 (11.3)	0.51	1
Diabetes onset age (years)	15.3 (8.8)	15.8 (9.1)	0.23	1
BMI (kg/m^2^)	24.7 (3.4)	25.3 (3.7)	< 0.001	< 0.001
Systolic blood pressure (mmHg)	133 (19)	136 (19)	< 0.001	0.004
Diastolic blood pressure (mmHg)	79 (10)	81 (11)	< 0.001	< 0.001
Retinal laser treatment (yes)	436 (36.4%)	503 (42.1%)	0.005	0.95
RAAS inhibitor (yes)	316 (26.4%)	440 (36.9%)	< 0.001	< 0.001
Lipid-lowering medication (yes)	91 (7.6%)	149 (12.5%)	< 0.001	0.002
Smoking status			< 0.001	< 0.001
Current	225 (19.5%)	322 (28.4%)		
Former	257 (22.3%)	266 (23.4%)		
Never	673 (58.3%)	547 (48.2%)		
HbA_1c_ (mmol/mol)	66.9 (15.9)	71.2 (16.1)	< 0.001	< 0.001
Total cholesterol (mmol/l)	4.86 (0.91)	5.19 (1.08)	< 0.001	< 0.001
HDL-cholesterol (mmol/l)	1.29 (0.35)	1.18 (0.35)	< 0.001	< 0.001
Triglycerides (mmol/l)	0.99 [0.77, 1.42]	1.23 [0.87, 1.81]	< 0.001	< 0.001
LDL-cholesterol (mmol/l)	3.03 (0.83)	3.33 (0.93)	< 0.001	< 0.001
DKD category at baseline			< 0.001	< 0.001
Normal AER	750 (62.2%)	662 (54.9%)		
Microalbuminuria	152 (12.6%)	161 (13.4%)		
Macroalbuminuria	182 (15.1%)	272 (22.6%)		
ESRD	121 (10.0%)	110 (9.1%)		
eGFR (ml/min/1.73 m^2^)	84 [67, 100]	83 [61, 99]	0.04	0.76
AER (mg/24 h)	11 [6, 41]	16 [7, 121]	< 0.001	< 0.001

Data are presented as mean (standard deviation), median [interquartile range], or n (%). *P*_*19 tests*_, p-value after Bonferroni correction for the total number of tests; BMI, body mass index; RAAS, renin–angiotensin–aldosterone system; HbA_1c_, glycated hemoglobin; HDL, high-density lipoprotein; LDL, low-density lipoprotein, DKD, diabetic kidney disease; AER, albumin excretion rate; ESRD, end-stage renal disease; eGFR, estimated glomerular filtration rat.

Furthermore, individuals in the category of higher H-ficolin had a poorer lipid profile compared to those in the other group, as also shown in Table 1. The associations between lipids and H-ficolin concentration were confirmed in additional correlation analyses, which revealed that the correlation with triglycerides was the strongest (r = 0.26, p < 0.001, *data not shown*).

### H-ficolin in relation to diabetic kidney disease

The mean H-ficolin concentration was higher with increasing stage of albumin excretion rate (AER) at baseline: 31.1 (10.0) µg/ml, 32.3 (10.6) µg/ml, and 33.9 (10.6) µg/ml in individuals with normal AER, microalbuminuria, and macroalbuminuria, respectively (p for trend < 0.001). Notably, individuals on dialysis had higher H-ficolin concentrations (41.2 [13.9] µg/ml, n = 69) than those with prior kidney transplantation (27.4 [9.2] µg/ml, n = 162), p < 0.001.

Of the 2,098 individuals with confirmed DKD status, 400 (19.1%) progressed during follow-up (141 [35.2%] to microalbuminuria, 60 [15.0%] to macroalbuminuria, 199 [49.8%] to end-stage renal disease, ESRD). The median [interquartile; IQR] follow-up time for the DKD outcomes was 7.9 [5.2, 13.4] years. With all events of progression pooled, the mean H-ficolin concentration was higher in those who progressed (34.5 [10.8] µg/ml) than those who did not (31.2 [10.0] µg/ml), p < 0.001. Likewise, among the individuals with initial normal AER and microalbuminuria, the mean H-ficolin concentration (µg/ml) was higher among progressors (34.2 [10.8] and 35.0 [10.3]) than non-progressors (30.1 [9.9] and 31.6 [10.3]), p < 0.001 and p = 0.02, respectively. A similar pattern, although non-significant, was seen in the individuals who progressed from macroalbuminuria to ESRD: 34.5 (11.0) vs. 33.5 (10.3) µg/ml, p = 0.29.

**Figure 1:**
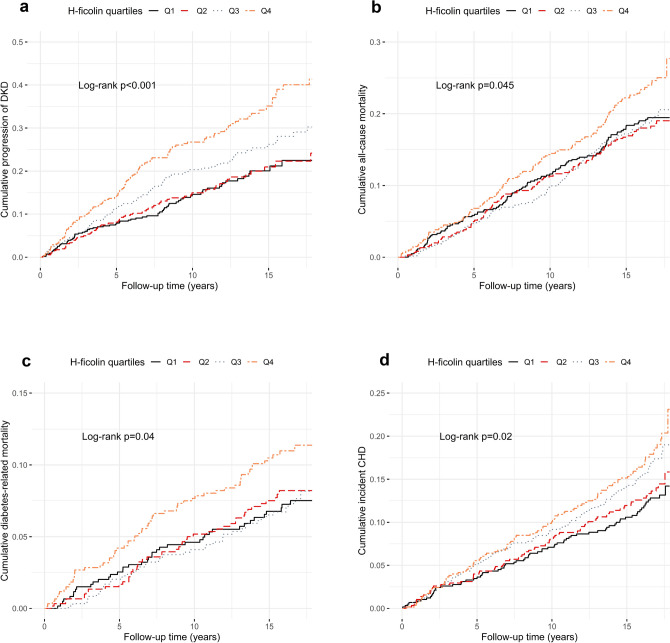
Kaplan–Meier curves, comparing individuals stratified by quartiles of H-ficolin concentration at baseline, with respect to (**a**) progression of diabetic kidney disease (DKD), (**b**) all-cause mortality, (**c**) diabetes-related mortality, and (**d**) coronary heart disease (CHD). Quartile 1 (Q1), black solid line; quartile 2 (Q2), red dashed line; quartile 3 (Q3), grey dotted line; quartile 4 (Q4), orange dot-dashed line.

The Kaplan–Meier plots in Fig. 1a illustrate the cumulative progression of DKD stratified by quartiles of H-ficolin concentration. The highest cumulative DKD progression was observed among those in the highest quartile of H-ficolin—while, as shown in the figure, the progression rate among the two lowest quartiles did not diverge.

Results from the Cox proportional hazard analyses exploring the relationship between H-ficolin and progression of DKD are presented in Table 2. The unadjusted hazard ratios (HRs) for a 10-unit increase of H-ficolin (all events of DKD progression pooled) was 1.29 (1.18–1.40), p < 0.001, and 1.16 (1.05–1.29), p = 0.003, after adjustment with diabetes duration, sex, HbA_1c_, systolic blood pressure, and history of smoking. However, in the final model further including triglycerides, the HR decreased to 1.07 (0.97–1.18), p = 0.19.

**Table 2:** Cox proportional hazards analysis with different levels of adjustment for DKD progression. Hazard ratios (HRs) are presented for a 10-unit increase in H-ficolin concentration.

	Number of individuals	Number of events	Model 1	Model 2	Model 3	**Model 4**
All events of progression	2,098	400	1.29 (1.18–1.40) p < 0.001	1.24 (1.13–1.35) p < 0.001	1.16 (1.05–1.29) p = 0.003	1.07 (0.97–1.18) p = 0.19
Normal AER to microalbuminuria	1,349	141	1.33 (1.16–1.53) p < 0.001	1.28 (1.11–1.48) p < 0.001	1.18 (1.00–1.39) p = 0.05	1.13 (0.93–1.34) p = 0.15
Micro- to macroalbuminuria	295	60	1.31 (1.04–1.64) p = 0.02	1.24 (0.97–1.59) p = 0.08	1.18 (0.91–1.52) p = 0.21	1.09 (0.84–1.42) p = 0.50
Macroalbuminuria to ESRD	454	199	1.10 (0.96–1.25) p = 0.16	1.09 (0.95–1.26) p = 0.20	1.10 (0.95–1.27) p = 0.22	1.00 (0.87–1.16) p = 0.97

Model 1: Unadjusted.

Model 2: Diabetes duration and sex.

Model 3: Model 2 + HbA_1c_, systolic blood pressure, and smoking status.

Model 4: Model 3 + triglycerides.

The HR for incident microalbuminuria was borderline significant after adjustment with diabetes duration, sex, HbA_1c_, systolic blood pressure, and history of smoking (1.18 [1.00–1.39]), p = 0.05, however, neither this association was independent of triglycerides (HR 1.13 [0.93–1.34], p = 0.15). Regarding progression to macroalbuminuria and ESRD, none of the multivariable analyses remained significant for H-ficolin, as shown in Table 2.

### H-ficolin in relation to mortality

A total of 486 participants died during follow-up. Regarding mortality, the individuals were followed for 16.5 (14.9–17.2) years. The mean concentration of H-ficolin was higher in those who died (33.0 [11.8] μg/ml) compared to those who did not (31.5 [10.1] μg/ml), p = 0.008. Altogether 268 deaths (55.1%) were due to cardiovascular causes and 192 (39.5%) to complications of diabetes. Of these, 63 deaths belonged to both groups. The mean H-ficolin concentration was no different (p = 0.52) stratified by cardiovascular mortality status, whereas individuals who died of diabetes-related causes had a higher mean H-ficolin (33.7 [12.1] μg/ml) than those who did not (31.7 [10.3] μg/ml), p = 0.008.

The highest quartile of H-ficolin also expressed the highest cumulative all-cause mortality as well as mortality from diabetes-related causes, as illustrated in Fig. 1b,c. Yet, there was no difference between the three lower quartiles regarding either of the outcomes (log-rank p-value 0.89 and 0.89).

Table 3 shows the different steps of Cox proportional hazards analyses for all-cause, cardiovascular, and diabetes-related mortality. Summarized, with the most stringent level of adjustment including diabetes duration, sex, HbA_1c_, systolic blood pressure, history of smoking, triglycerides, and DKD category of baseline (Model 5), only the association between H-ficolin and diabetes-related mortality remained significant (HR 1.18 [1.02–1.37], p = 0.02). As the cumulative incidences for all-cause and diabetes-related mortality were the highest for quartile 4 (Q4), we also performed the Cox regression analyses comparing Q4 with Q1-3 combined. Adjusting for Model 5, the HR for Q4 regarding all-cause mortality was 1.26 (1.02–1.56), p = 0.03, and 1.62 (1.15–2.27), p = 0.005, regarding mortality attributable to complications of diabetes.

**Table 3:** Cox proportional hazards analysis with different levels of adjustment for cardiovascular outcomes and mortality. Hazard ratios (HRs) are presented for a 10-unit increase in H-ficolin concentration.

	Number of individuals	Number of events	Model 1	Model 2	Model 3	Model 4	Model 5
All-cause mortality	2,410	486	1.13 (1.04–1.22) p = 0.005	1.11 (1.01–1.21) p = 0.02	1.09 (0.99–1.20) p = 0.08	1.01 (0.92–1.11) p = 0.83	1.06 (0.97–1.17) p = 0.19
Cardiovascular mortality	2,410	268	1.05 (0.93–1.17) p = 0.43	1.03 (0.91–1.16) p = 0.68	1.00 (0.88–1.14) p = 0.98	0.92 (0.80–1.05) p = 0.20	0.98 (0.86–1.12) p = 0.79
Diabetes-related mortality	2,410	192	1.19 (1.05–1.35) p = 0.008	1.22 (1.06–1.40) p = 0.005	1.22 (1.05–1.42) p = 0.009	1.11 (0.95–1.29) p = 0.20	1.18 (1.02–1.37) p = 0.02
CHD	2,311	354	1.15 (1.05–1.26) p = 0.003	1.13 (1.03–1.25) p = 0.01	1.10 (1.00–1.22) p = 0.06	1.05 (0.95–1.16) p = 0.34	1.06 (0.96–1.16) p = 0.28
Stroke	2,357	176	1.08 (0.95–1.24) p = 0.24	1.03 (0.90–1.19) p = 0.64	1.01 (0.87–1.17) p = 0.88	0.98 (0.84–1.14) p = 0.79	1.00 (0.86–1.15) p = 0.99

Model 1: Unadjusted.

Model 2: Diabetes duration and sex.

Model 3: Model 2 + HbA_1c_, systolic blood pressure, and smoking status.

Model 4: Model 3 + triglycerides.

Model 5: Model 4 + DKD category at baseline.

### H-ficolin in relation to cardiovascular events

For CHD and stroke, the median [IQR] follow-up time was 16.7 [15.2, 17.3] and 16.9 [15.6, 17.3] years, respectively. The individuals who experienced an incident CHD event were characterized by a higher mean H-ficolin concentration (33.4 [10.3]) than their counterparts who did not (31.6 [10.5]), p = 0.003. However, we did not observe a difference in H-ficolin concentration stratified by incident stroke (32.7 [10.5] vs. 31.6 [10.5]), p = 0.24.

The cumulative incidence of CHD increased stepwise with increasing quartile of H-ficolin concentration, as Fig. 1d shows (log-rank p = 0.02). The HR for a 10-unit increase in H-ficolin concentration was 1.15 (1.05–1.26), p = 0.003 in unadjusted analysis, and 1.13 (1.03–1.25), p = 0.01, after adjustment for diabetes duration and sex. However, this association was not significant after further adjustments (Table 3). As presumed from the univariate analysis, the Cox regressions yielded no significant associations between H-ficolin and stroke (Table 3).

## Discussion

Findings from experimental and clinical studies are pointing towards inflammation and the complement system contributing to the pathogenesis of diabetic complications, particularly DKD. H-ficolin is a pattern-recognition molecule that can activate the lectin pathway of the complement system by recognition of specific carbohydrate or acetyl-patterns on the surface of microorganisms or altered self-cells^17,18^. The lectin pathway targets complement activation based on the reaction between pattern-recognition molecules (including H-ficolin) and so-called pathogen-associated molecular patterns (PAMPs) on non-self surfaces, as well as damage- or danger-associated molecular patterns (DAMPs) on self-cells, such as apoptotic cells. In the Steno Diabetes cohort, we previously found that circulating H-ficolin concentration is associated with incident microalbuminuria in individuals with newly diagnosed type 1 diabetes, independent of HbA_1c_, systolic blood pressure, smoking, and baseline urinary AER^4^. In the present study, we aimed to investigate this relationship in further detail in a larger cohort with more events, including progression to the more advanced stages of DKD. In a cross-sectional setting, we observed increasing H-ficolin concentrations with a more advancing level of DKD at baseline. Even though H-ficolin was associated with the pooled outcome of DKD progression as well as incident microalbuminuria separately, these associations lost statistical significance in the final model that adjusted for triglycerides in addition to diabetes duration, sex, HbA_1c_, systolic blood pressure, and smoking (*i.e.,* traditional risk factors for DKD progression and the other outcomes of the study). H-ficolin was not associated with the incidence of macroalbuminuria or ESRD. Although the low number of progressors from micro- to macroalbuminuria and ESRD may be a contributing factor, our observational study setting does not explain this discrepancy.

MBL is the classic activator of the lectin pathway of the complement system and has been linked to DKD as well as all-cause mortality in both type 1 and type 2 diabetes^5–9,19^. One of the main aims of our study was to add to this knowledge by assessing the association between the lectin pathway activator H-ficolin and mortality in type 1 diabetes. We found that individuals in the highest quartile of H-ficolin had the highest all-cause mortality during follow-up, as compared to the individuals with lower H-ficolin levels. However, even though an association between H-ficolin concentration and all-cause mortality was seen in the present study, it was not statistically significant in the adjusted analyses. On one hand, these results may reflect stronger predictive values of the covariates including HbA_1c_, blood pressure, smoking, triglycerides, and AER as compared with H-ficolin. On the other hand, the covariates may be confounders but also a step on a causal pathway between H-ficolin and mortality. Mutations in the H-ficolin encoding *FCN3* gene are rare and, therefore, it remains to be determined whether H-ficolin concentration per se may cause alterations in the covariates of our statistical models^20,21^. This is of special interest as a correlation has been observed between the triglycerides and H-ficolin, both in this study and by others^22,23^.

Cardiovascular, along with endocrine, causes have been the most frequently reported contributors to death in several cohorts of individuals with type 1 diabetes^24–27^. In line, the majority (55%) of deaths in this population were due to cardiovascular disease (CVD). We did not observe any difference in the H-ficolin concentration between individuals who died from CVD causes as compared with those who did not, and moreover, the H-ficolin level did not predict CHD events or stroke in the adjusted models. Previous data on H-ficolin in CVD are sparse. H-ficolin level did not differ in a cross-sectional study of healthy subjects and patients with myocardial infarction^28^. In ischemic stroke, a cross-sectional study found H-ficolin level to be inversely correlated to disease severity, which was speculated to be a consequence of H-ficolin binding to dying cells^29^. More data exists for MBL in relation to CVD, in which MBL seems to act as a double-edged sword. In stroke and myocardial infarction, MBL is found to augment tissue damage most likely through reaction with altered self-surfaces whereas beneficial effects of MBL in tissue homeostasis are observed in the development of atherosclerotic lesions^30^. Yet, although diabetic micro- and macrovascular complications tend to coincide, the present findings do not identify H-ficolin as the link.

One of the most intriguing findings from the present study was related to mortality due to complications of diabetes, an outcome confirmed from both register-based data and death certificates by FinnDiane researchers. In contrast to cardiovascular mortality, we found a robust association with all analyses, and the HR for diabetes-related death for every 10-unit increase in H-ficolin concentration was 1.18-fold despite the most stringent level of adjustment, including baseline DKD stage. Previous studies from both the FinnDiane as well as other cohorts have established that the presence and the severity of renal complications is the main predictor of survival in type 1 diabetes^31–33^. Notably, acute complications such as hypoglycemia or diabetic ketoacidosis constituted only a minority of the cases in our diabetes-related outcome*,* whereas *insulin-dependent diabetes mellitus with multiple complications* was the most frequently reported underlying cause of death.

The possible mechanistic pathway underlying the observed association has not been unraveled yet—however, we speculate that one explanation could be tissue remodeling and damage caused by diabetes. The terminal membrane-bound product of the complement cascade is the membrane-attack complex (MAC), which is a transmembrane pore that causes lysis of invading pathogens by osmotic stress. Healthy mammalian cells are normally protected from complement auto-attack by complement regulatory proteins that inhibit the complement cascade, for instance, CD59. However, hyperglycemia-induced glycation of CD59 in diabetes impairs the protection from complement auto-attack^34,35^. MAC-targeted endothelium cells have been found to stimulate mitosis of glomerular mesangial cells, which further supports the view^36^.

The results of the present study need to be interpreted with attention to design strengths and weaknesses. The observational design prevents inference of causality. Also, the cumulative numbers of specific events may be too low to avoid statistical type 2 errors even in this large cohort of individuals with long observation time. However, the cohort is very well characterized and includes patients from multiple centers throughout Finland. Likewise, complete register-based coverage of cardiovascular events and mortality in addition to AER from the review of medical records are strengths of the study.

In conclusion, this study shows that high concentrations of serum H-ficolin predict mortality attributable to diabetes in adjusted analyses, whereas an association between H-ficolin and all-cause mortality was not observed in our cohort. No link between H-ficolin and cardiovascular events was detected, and despite that the association with DKD progression was independent of several well-established risk factors, it did not persist after adjustment with triglycerides. These results add to the existing knowledge of the complement system in long-term complications of type 1 diabetes. Yet, our findings stem from an observational study setting, and therefore, more research is needed to investigate possible causalities between H-ficolin and diabetes-related mortality.

## Methods

### Study population

The FinnDiane Study is a prospective, observational cohort study recruiting adult individuals with type 1 diabetes from multiple centers throughout Finland with the aim to detect risk factors for late diabetic complications in type 1 diabetes. The study was initiated in 1997 and the follow-up protocol has previously been described in detail^37^. Only individuals with established type 1 diabetes have been recruited to the FinnDiane Study (International Classification of Diseases [tenth revision; ICD-10] classification code E10 as a prerequisite), but as proof of a correct diagnosis, the subjects included in the present study were under the age of 40 years at diabetes onset and their insulin treatment was initiated within the first year from the diabetes diagnosis.

The participants underwent a thorough clinical examination at baseline. Information on co-morbidities and medication were obtained through questionnaires accompanied by drawing of blood samples and timed overnight or 24-h urine collections. HbA_1c_ was measured using standardized assays at the local diabetes centers. Smoking status was defined as current if the study participant had smoked at least one cigarette per day for at least one year. Participants who had stopped smoking before entering the FinnDiane study were considered former smokers. Kidney function was estimated by the Chronic Kidney Disease Epidemiology Collaboration (CKD-EPI) formula^38^. Patients with undetermined DKD status at baseline (n = 68) were excluded from the analyses.

All study participants gave their written informed consent before inclusion. The study was conducted following the Declaration of Helsinki and approved by the Ethics Committee of the Helsinki and Uusimaa Hospital District.

### Outcomes

Follow-up data on kidney outcomes are recorded continuously for participants in the FinnDiane study. AER was categorized as normal range (AER < 20 µg/min or < 30 mg/24 h or albumin-creatinine ratio [ACR] < 2.5 mg/mmol for men or < 3.5 mg/mmol for women), microalbuminuria (AER ≥ 20 and < 200 µg/min or ≥ 30 and < 300 mg/24 h or ACR ≥ 2.5 and < 25 mg/mmol for men or ≥ 3.5 and < 35 mg/mmol for women), or macroalbuminuria (AER ≥ 200 µg/min or ≥ 300 mg/24 h or ACR > 25 mg/mmol for men or > 35 mg/mmol for women). ESRD was defined as an ongoing need for dialysis or kidney transplantation. The most advanced DKD stage in two out of three consecutive urine collections preceding baseline was taken as the basis for the classification. Progression of DKD was defined as a change to a more advanced stage of AER or initiation of renal-replacement therapy, confirmed from the medical files.

The analyses of cardiovascular events and mortality included events tracked through December 31, 2015. Non-fatal CVD events were retrieved from the Finnish National Care Register for Health Care, whereas the events of death were retrieved from the Finnish Cause of Death Register maintained by Statistics Finland. Only incident cases of CHD and stroke were included, hence, the study participants with events before the study baseline were excluded from these analyses. Death certificates were available for 98.8% of the deceased study participants. Cause of death was based on information reviewed from these by L.M.T., as well as on ICD-10 codes provided by Statistics Finland. Mortality was defined as cardiovascular if the underlying and/or immediate cause of death was due to cardiovascular causes (ICD-10 I00-I99), and as diabetes-related if the cause was due to acute or chronic complications of the disease. The underlying cause of the diabetes-related death was chronic diabetes complications in 91.7% of the cases (n = 176) and acute complications in 8.3% (n = 16). Of the 176 chronic complications, 75.0% were coded as E10.7 (*Type 1 diabetes mellitus with multiple complications*) and 14.8% as E10.2 (*Type 1 diabetes mellitus with kidney complications).* The immediate causes of diabetes-related deaths were CVD (31.8%, n = 61), infections (28.1%, n = 54), chronic diabetes complications (22.9%, n = 44), and acute diabetes complications (10.9%, n = 21). The remaining causes were unclear (6.3%, n = 12) and, therefore, categorized only based on the underlying cause. Events of coronary heart disease (CHD) were defined as cases of myocardial infarction (ICD-10 121-I22, ICD-8/9 410), coronary artery bypass grafting, and percutaneous coronary intervention (procedure codes TFN40, FN1AT, FN1BT, FN1YT, FNF, FNG, FNA, FNB, FNC, FND, FNE since 1996 and 5311–5315 between the years 1983 and 1995).

### H-ficolin

Quantification of the H-ficolin concentration was performed blinded to subject identity as previously described using normal human serum as standard except for a few changes^39^. In brief, serum was thawed, diluted in assay buffer, and added to microtiter wells (FluoroNunc, Thermo Scientific, Waltham, MA, USA) coated with acetylated bovine serum albumin (B2518, Sigma-Aldrich, St. Louis, MO, USA), which is recognized by H-ficolin. Standards, samples, and controls were added automatically to plates using a Janus Varispan automated work-station (PerkinElmer, Waltham, MA, USA). In-house biotinylated anti-H-ficolin antibody, europium-labelled streptavidin (PerkinElmer) and enhancement solution (Ampliqon, Odense, Denmark) were added in successive steps with triple washing in between. The europium fluorescence intensity was detected with a Victor X5 fluorometer (PerkinElmer). Intra-assay and inter-assay coefficients of variation were below 10% and 16%, respectively. H-ficolin concentration was used as a predictor as a continuous variable as well as by comparing subjects grouped according to H-ficolin quartiles.

### Statistical analysis

Continuous variables were compared with Student’s t-test (two groups) or ANOVA (several groups) and expressed as mean (standard deviation; SD) if the data were normally distributed or with Mann–Whitney U-test and expressed as median [IQR] if the data distribution was skewed. Categorical variables were expressed as n (%) and compared between the groups using χ^2^ test. Multiple testing was addressed by Bonferroni correction. There was no interaction between H-ficolin and sex for any of the outcomes, hence, men and women were pooled in the analyses. Time-to-event was illustrated by Kaplan–Meier plots and the log-rank test was used to compare survival distributions between quartiles of H-ficolin concentration. In addition, Cox proportional hazards analyses were used to estimate the HRs after adjustment for risk factors. The HRs are presented with 95% confidence interval for a 10-unit increase in H-ficolin concentration. The relationship between H-ficolin and components of the lipid profile were assessed with correlation analyses. Data analysis was performed with R open-source software version 3.5.1 (http://r-project.org). Two-sided p-values below 5% were considered statistically significant.

## Supplementary Information

Supplementary Information.
