# Supplementary material for: The pattern-recognition molecule H-ficolin in relation to diabetic kidney disease, mortality, and cardiovascular events in type 1 diabetes
Source: Sci Rep. 2021 Apr 26;11:8919. doi: 10.1038/s41598-021-88352-y (PMC8076270; doi:10.1038/s41598-021-88352-y)
Supplement: Supplementary file 1 — Supplementary Information. [file 41598_2021_88352_MOESM1_ESM.pdf]

The pattern-recognition molecule H-ficolin in relation to diabetic kidney disease, mortality, and cardiovascular events in type 1 diabetes

Jakob Appel Østergaard [†1,2], Fanny Jansson Sigfrids[†3-5], Carol Forsblom[3-5], Emma H Dahlström[3-5], Lena M Thorn[3-6], Valma Harjutsalo[3-5,9], Allan Flyvbjerg[7], Steffen Thiel[8], Troels Krarup Hansen[2], and Per-Henrik Groop[3-5,10]

[†] = equal contribution

Corresponding author: Per-Henrik Groop, Biomedicum Helsinki (C318b), Haartmaninkatu 8, FIN-00290 Helsinki, Finland, +358-50-0430436, per-henrik.groop@helsinki.fi, ORCID iD: https://orcid.org/0000-0003-4055-6954)

[1]Department of Endocrinology and Internal Medicine, Aarhus University Hospital, Aarhus, Denmark

[2]Steno Diabetes Center Aarhus, Aarhus University Hospital, Aarhus, Denmark

[3]Folkhälsan Institute of Genetics, Folkhälsan Research Center, Helsinki, Finland [4]Abdominal Center, Nephrology, University of Helsinki and Helsinki University Hospital, Helsinki, Finland

[5]Research Program for Clinical and Molecular Metabolism, Faculty of Medicine, University of Helsinki, Helsinki, Finland

[6]Department of General Practice and Primary Health Care, University of Helsinki and Helsinki University Hospital, Helsinki, Finland

[7]Steno Diabetes Center Copenhagen, The Capital Region of Denmark, Copenhagen, Denmark

[8]Department of Biomedicine, Aarhus University, Aarhus, Denmark

[9]National Institute for Health and Welfare, Helsinki, Finland

[10]Department of Diabetes, Central Clinical School, Monash University, Melbourne, Victoria, Australia.

Supplementary Table S1 Physicians and nurses at health care centers participating in the collection of FinnDiane patients.

**FinnDiane Study Centers**	**Physicians and nurses**
Anjalankoski Health Centre	S. Koivula, T. Uggeldahl
Central Finland Central Hospital, Jyväskylä	T. Forslund, A. Halonen, A. Koistinen, P. Koskiaho, M. Laukkanen, J. Saltevo, M. Tiihonen
Central Hospital of Åland Islands, Mariehamn	M. Forsen, H. Granlund, A-C. Jonsson, B. Nyroos
Central Hospital of Kanta-Häme, Hämeenlinna	P. Kinnunen, A. Orvola, T. Salonen, A. Vähänen
Central Hospital of Länsi-Pohja, Kemi	H. Laukkanen, P. Nyländen, A. Sademies
Central Ostrabothnian Hospital District, Kokkola	S. Anderson, B. Asplund, U. Byskata, P. Liedes, M. Kuusela, T. Virkkala
City of Espoo Health Centre	
Espoonlahti	A. Nikkola, E. Ritola
Tapiola	M. Niska, H. Saarinen
Samaria	E. Oukko-Ruponen, T. Virtanen
Viherlaakso	A. Lyytinen
City of Helsinki Health Centre	
Puistola	H. Kari, T. Simonen
Suutarila	A. Kaprio, J. Kärkkäinen, B. Rantaeskola
Töölö	P. Kääriäinen, J. Haaga, A-L. Pietiläinen
City of Hyvinkää Health Centre	S. Klemetti, T. Nyandoto, E. Rontu, S. Satuli-Autere
City of Vantaa Health Centre	
Korso	R. Toivonen, H. Virtanen
Länsimäki	R. Ahonen, M. Ivaska-Suomela, A. Jauhiainen
Martinlaakso	M. Laine, T. Pellonpää, R. Puranen
Myyrmäki	A. Airas, J. Laakso, K. Rautavaara
Rekola	M. Erola, E. Jatkola
Tikkurila	R. Lönnblad, A. Malm, J. Mäkelä, E. Rautamo
Heinola Health Centre	P. Hentunen, J. Lagerstam
Helsinki University Central Hospital, Department of Medicine, Division of Nephrology	A. Ahola, J. Fagerudd, M. Feodoroff, D. Gordin, O. Heikkilä, K Hietala, L. Kyllönen, J. Kytö, S. Lindh, K. Pettersson-Fernholm, M. Rosengård-Bärlund, M. Rönnback, A. Sandelin, A-R Salonen, L. Salovaara, L. Thorn, J. Tuomikangas, T. Vesisenaho, J. Wadén
Herttoniemi Hospital, Helsinki	V. Sipilä

FinnDiane Study Centers	Physicians and nurses
Hospital of Lounais-Häme, Forssa	T. Kalliomäki, J. Koskelainen, R. Nikkanen, N. Savolainen, H. Sulonen, E. Valtonen
Iisalmi Hospital	E. Toivanen
Jokilaakso Hospital, Jämsä	A. Parta, I. Pirttiniemi
Jorvi Hospital, Helsinki University Central Hospital	S. Aranko, S. Ervasti, R. Kauppinen-Mäkelin, A. Kuusisto, T. Leppälä, K. Nikkilä, L. Pekkonen
Jyväskylä Health Centre, Kyllö	K. Nuorva, M. Tiihonen
Kainuu Central Hospital, Kajaani	S. Jokelainen, P. Kemppainen, A-M. Mankinen, M. Sankari
Kerava Health Centre	H. Stuckey, P. Suominen
Kirkkonummi Health Centre	A. Lappalainen, M. Liimatainen, J. Santaholma
Kivelä Hospital, Helsinki	A. Aimolahti, E. Huovinen
Koskela Hospital, Helsinki	V. Ilkka, M. Lehtimäki
Kotka Heath Centre	E. Pälikkö-Kontinen, A. Vanhanen
Kouvola Health Centre	E. Koskinen, T. Siitonen
Kuopio University Hospital	E. Huttunen, R. Ikäheimo, P. Karhapää, P. Kekäläinen, M. Laakso, T. Lakka, E. Lampainen, L. Moilanen, L. Niskanen, U. Tuovinen, I. Vauhkonen, E. Voutilainen
Kuusamo Health Centre	T. Kääriäinen, E. Isopoussu
Kuusankoski Hospital	E. Kilkki, I. Koskinen, L. Riihelä
Laakso Hospital, Helsinki	T. Meriläinen, P. Poukka, R. Savolainen, N. Uhlenius
Lahti City Hospital	A. Mäkelä, M. Tanner
Lapland Central Hospital, Rovaniemi	L. Hyvärinen, S. Severinkangas, T. Tulokas
Lappeenranta Health Centre	P. Linkola, I. Pulli
Lohja Hospital	T. Granlund, M. Saari, T. Salonen
Loimaa Health Centre	A. Mäkelä, P. Eloranta
Länsi-Uusimaa Hospital, Tammisaari	I-M. Jousmaa, J. Rinne
Malmi Hospital, Helsinki	H. Lanki, S. Moilanen, M. Tilly-Kiesi
Mikkeli Central Hospital	A. Gynther, R. Manninen, P. Nironen, M. Salminen, T. Vänttinen
Mänttä Regional Hospital	I. Pirttiniemi, A-M. Hänninen
North Karelian Hospital, Joensuu	U-M. Henttula, P. Kekäläinen, M. Pietarinen, A. Rissanen, M. Voutilainen
Nurmijärvi Health Centre	A. Burgos, K. Urtamo
Oulankangas Hospital, Oulainen	E. Jokelainen, P-L. Jylkkä, E. Kaarlela, J. Vuolaspuro
Oulu Health Centre	L. Hiltunen, R. Häkkinen, S. Keinänen-Kiukaanniemi
Oulu University Hospital	R. Ikäheimo
Päijät-Häme Central Hospital	H. Haapamäki, A. Helanterä, S. Hämäläinen, V. Ilvesmäki, H. Miettinen

FinnDiane Study Centers	Physicians and nurses
Palokka Health Centre	P. Sopanen, L. Welling
Pieksämäki Hospital	V. Javtsenko, M. Tamminen
Pietarsaari Hospital	M-L. Holmbäck, B. Isomaa, L. Sarelin
Pori City Hospital	P. Ahonen, P. Merensalo, K. Sävelä
Porvoo Hospital	M. Kallio, B. Rask, S. Rämö
Raahe Hospital	A. Holma, M. Honkala, A. Tuomivaara, R. Vainionpää
Rauma Hospital	K. Laine, K. Saarinen, T. Salminen
Riihimäki Hospital	P. Aalto, E. Immonen, L. Juurinen
Salo Hospital	A. Alanko, J. Lapinleimu, P. Rautio, M. Virtanen
Satakunta Central Hospital, Pori	M. Asola, M. Juhola, P. Kunelius, M-L. Lahdenmäki, P. Pääkkönen, M. Rautavirta
Savonlinna Central Hospital	E. Korpi-Hyövälti, T. Latvala, E. Leijala
South Karelia Central Hospital, Lappeenranta	T. Ensala, E. Hussi, R. Härkönen, U. Nyholm, J. Toivanen
Tampere Health Centre	A. Vaden, P. Alarotu, E. Kujansuu, H. Kirkkopelto-Jokinen, M. Helin, S. Gummerus, L. Calonius, T. Niskanen, T. Kaitala, T. Vatanen
Tampere University Hospital	I. Ala-Houhala, T. Kuningas, P. Lampinen, M. Määttä, H. Oksala, T. Oksanen, K. Salonen, H. Tauriainen, S. Tulokas
Tiirismaa Health Centre, Hollola	T. Kivelä, L, Petlin, L. Savolainen
Turku Health Centre	I. Hämäläinen, H. Virtamo, M. Vähätalo
Turku University Central Hospital	K. Breitholz, R. Eskola, K. Metsärinne, U. Pietilä, P. Saarinen, R. Tuominen, S. Äyräpää
Vaajakoski Health Centre	K. Mäkinen, P. Sopanen
Valkeakoski Regional Hospital	S. Ojanen, E. Valtonen, H. Ylönen, M. Rautiainen, T. Immonen
Vammala Regional Hospital	I. Isomäki, R. Kroneld, M. Tapiolinna-Mäkelä
Vaasa Central Hospital	S. Bergkulla, U. Hautamäki, V-A. Myllyniemi, I. Rusk
